# Natural variation of hormone levels in *Arabidopsis* roots and correlations with complex root architecture

**DOI:** 10.1111/jipb.12617

**Published:** 2018-02-06

**Authors:** Sangseok Lee, Lidiya I. Sergeeva, Dick Vreugdenhil

**Affiliations:** ^1^ Laboratory of Plant Physiology Wageningen University & Research Droevendaalsesteeg 1 6708 PB Wageningen The Netherlands; ^2^ Gyeongsangbuk‐Do Agricultural Research & Extension Services Centre 136 Gil‐14 Chilgokjungang‐daero Daegu South Korea

## Abstract

Studies on natural variation are an important tool to unravel the genetic basis of quantitative traits in plants. Despite the significant roles of phytohormones in plant development, including root architecture, hardly any studies have been done to investigate natural variation in endogenous hormone levels in plants. Therefore, in the present study a range of hormones were quantified in root extracts of thirteen *Arabidopsis thaliana* accessions using a ultra performance liquid chromatography triple quadrupole mass spectrometer. Root system architecture of the set of accessions was quantified, using a new parameter (mature root unit) for complex root systems, and correlated with the phytohormone data. Significant variations in phytohormone levels among the accessions were detected, but were remarkably small, namely less than three‐fold difference between extremes. For cytokinins, relatively larger variations were found for ribosides and glucosides, as compared to the free bases. For root phenotyping, length‐related traits—lateral root length and total root length—showed larger variations than lateral root number‐related ones. For root architecture, antagonistic interactions between hormones, for example, indole‐3‐acetic acid to *trans‐*zeatin were detected in correlation analysis. These findings provide conclusive evidence for the presence of natural variation in phytohormone levels in *Arabidopsis* roots, suggesting that quantitative genetic analyses are feasible.

**Edited by:** Wolfgang Busch, Salk Institute for Biological Studies, USA

## INTRODUCTION

As sessile organisms, plants have to respond adequately to the environment into which they are growing. Environmental cues are perceived by plants and translated into internal signals, including phytohormones. These hormones, alone or in interactions, regulate the growth and development of the plant, ensuring optimal survival and reproduction. Within a species, natural variation occurs for many traits, due to adaptation of populations to local environments (Koornneef et al. 2004; Alonso‐Blanco et al. 2009). Natural accessions and sets of recombinant inbred lines (RIL), derived from such ecotypes, vary considerably for primary and secondary metabolite contents, such as for instance flavonoids in *Arabidopsis* seeds (Kliebenstein et al. 2001; Routaboul et al. 2012). It thus seems logical to assume that natural variation will also be present for hormone contents in plants. Although some reports describe variation for the response to exogenous hormone application (Pilet and Saugy 1987; Kliebenstein et al. 2001; Novakova et al. 2005; Kanno et al. 2010; Dobon et al. 2011) and for hormone levels in leaves and root exudates (Cardoso et al. 2014; Monchgesang et al. 2016; Nam et al. 2017), data on natural variation in endogenous hormone concentrations in *Arabidopsis* roots are absent.

Root initiation and branching are well‐known examples of plant developmental processes, mediated by plant hormones. Lateral root initiation is controlled by hormone interactions (Tanimoto 2005; Garay‐Arroyo et al. 2012). Specifically, cytokinins (CKs) and auxin are key phytohormones that regulate root development, vascular differentiation and gravitropism (Aloni et al. 2006). The observed auxin maximum in the root apical region, resulting from specific cellular localization of auxin efflux carriers (PIN proteins), suggests that an indole‐3‐acetic acid (IAA) gradient plays a significant role in the dynamics of root growth (Tanimoto 2005; Petersson et al. 2009). CKs, acting as negative regulators of lateral root initiation, are known to perturb the auxin gradient by controlling PIN‐dependent transport (Laplaze et al. 2007; Marhavy et al. 2011; Marhavy et al. 2014). Recently, concentration gradients for different CK metabolites were detected in the primary root zones and different cells/tissues (Bielach et al. 2012; Antoniadi et al. 2015).

Despite a wealth of studies aimed at unravelling biological functions of phytohormones in various root developmental processes, at least the following points have yet to be answered, and will be the focus of the present study. (i) Is natural variation present for hormone contents, and if so, to what extent? (ii) How does this natural variation of phytohormone levels correlate with root system architecture (RSA)?

Numerous recent studies have been published addressing effective methods for quantitative high‐throughput root phenotyping, coupled with computational software (Iyer‐Pascuzzi et al. 2010; De Smet et al. 2012; Clark et al. 2013; Slovak et al. 2014). In order to describe and quantify root traits in dicotyledonous species, for instance *Arabidopsis thaliana*, most descriptive traits are determined by two key components—the primary root and the lateral roots—concerning both lengths and numbers (Smith and De Smet 2012; Tian et al. 2014).

Up to now, most studies on RSA in *Arabidopsis* have been conducted at seedling stages to avoid the complexity of measuring root traits in mature plants (Dubrovsky and Forde 2012). The vertical agar plate, a common *in vitro* culture method for *Arabidopsis*, results in two‐dimensional patterns of root growth and allows relatively easy scanning of roots. Such root phenotypings have been conducted exclusively on seedlings—younger than 2 weeks after germination—because of the space limitation in Petri dishes and difficulties in separating each individual root from others in case of a complex root system. Little attention has been given to more mature roots (older than 3 weeks), or root traits beyond the seedling stages.

Analyzing the natural variation in phytohormone levels will be an important tool to increase our understanding of the genetic variation governing the molecular mechanisms of hormonal regulation in developmental processes (Korstanje and Paigen 2002). Here, we report the variation in endogenous phytohormone levels in roots of 13 *A. thaliana* accessions. Simultaneously, the RSA was analyzed using a new descriptive phenotypic trait, coined mature root unit (MRU), as a descriptive developmental unit. Our finding that natural variation in root phytohormones in *Arabidopsis* exists, suggests that quantitative genetic approaches are feasible to dissect the molecular elements responsible for phytohormone levels and their effect on changes in root architecture.

## RESULTS

### Accessions and hydroponic culture

Thirteen accessions were selected based on genetic variation (Horton et al. 2012), and on earlier observed variations in growth patterns and primary metabolites (El‐Lithy et al. 2010). The set of accessions largely overlapped with collections used for natural variation studies of various traits (McKhann et al. 2004; Sutka et al. 2011). The first objective was to develop a reliable method of cultivation, in order to uniformly grow roots, suitable for phenotyping, and yielding sufficient biomass for the hormone analyses. Eppendorf tubes (0.5 mL), of which the tip and the lid were removed, were filled with 0.5% agar in half strength Hoagland's solution and used as seed support. Roots of seedlings easily grew through the agar and subsequently reached the hydroponic solution underneath (Figure S1). In comparison with spray‐type aeroponics, hydroponics turned out to be more suitable for uniform root development (data not shown).

**Figure 1:**
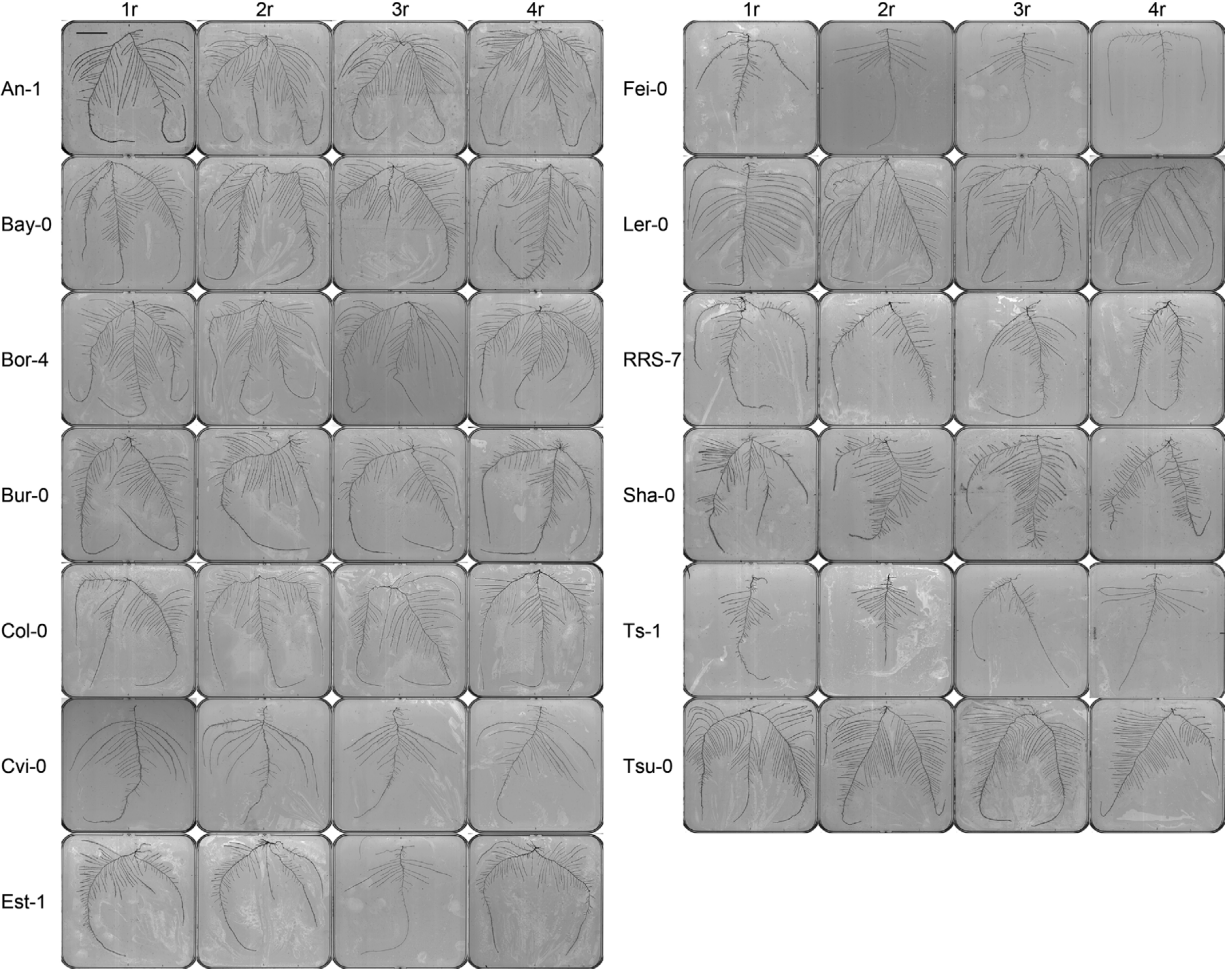
**2D‐root images of 13 *Arabidopsis* accessions after 23 d of culture on hydroponics** From left to right, four replicates of each accession are presented. At the upper‐left corner in the first replicate of An‐1, the length of scale bar is 3 cm.

In order to extend the period of vegetative development and to avoid physiological changes due to initiation of flowering, plants were grown under short days. Under the applied culture conditions, none of the 13 accessions showed visible signs of floral transition before 40 d after germination. In a more extended time‐course experiment with Columbia‐0 (Col‐0) and Landsberg erecta (Ler‐0), no sign of flowering was observed until 62 d. Twenty‐three‐d‐old plants were used as they have a root system with multiple‐order branching, but were still suitable for two‐dimensional root phenotyping. At that time point, the accessions displayed a wide diversity in root architecture (Figure 1).

### Parameters to quantify root traits

**Figure 2:**
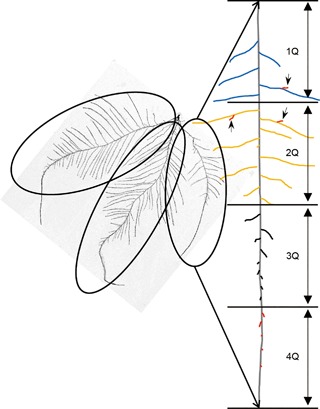
**Schematic concept of mature root unit (MRU) and the quarterly distribution of lateral roots** In this example image, the root system consists of three MRUs, indicated by the ellipses. A MRU on the right side is magnified in a linear way. Lateral roots alongside the main root axis are colored differently (blue, yellow, black, red) in four quarter panels from root base (1Q) to root tip (4Q). Secondary lateral roots (2′‐LR, orange color, indicated by short arrows) are roots originating from lateral roots in main root units.

**Figure 3:**
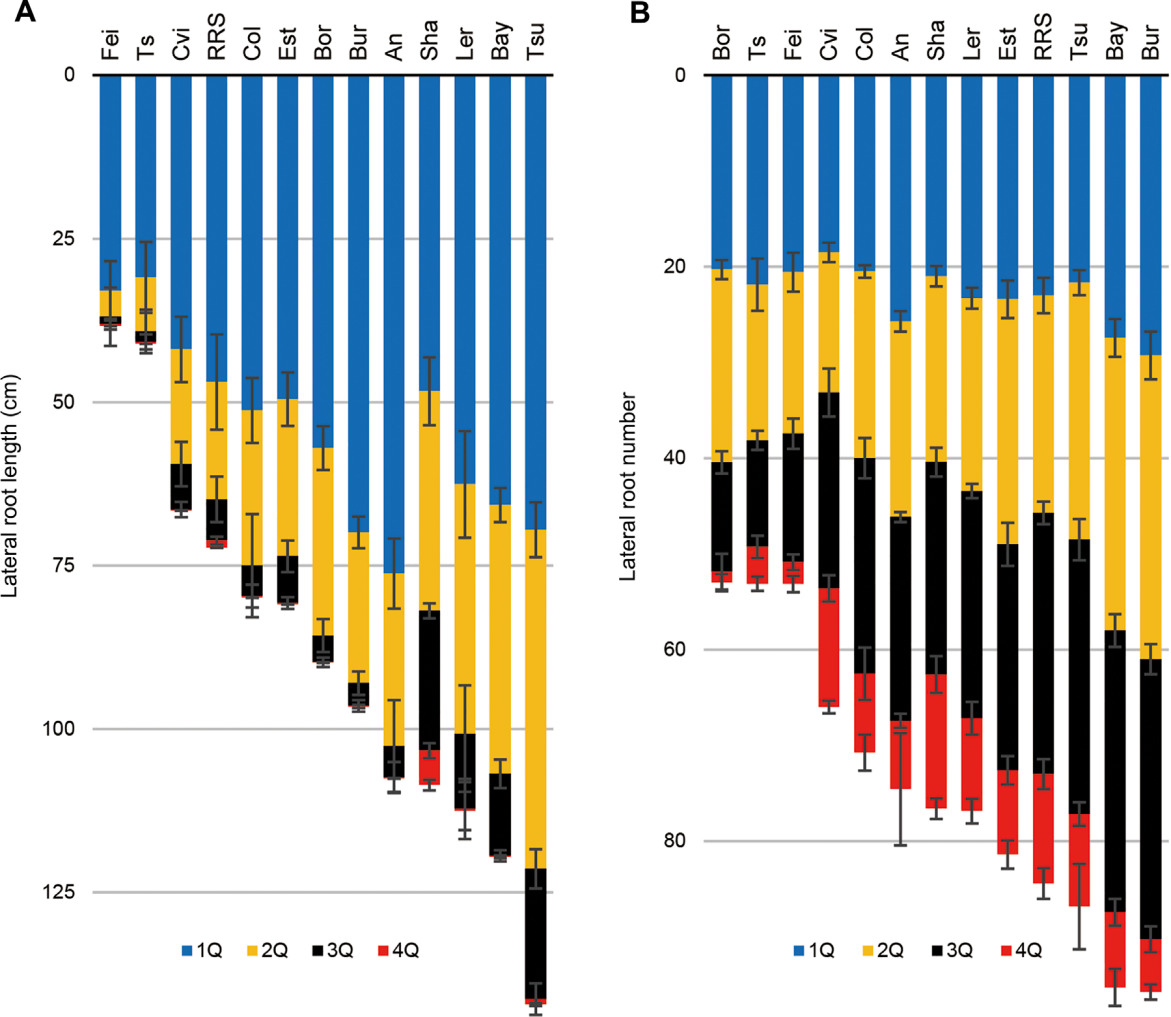
**Quarterly distribution of lateral root length and lateral root number in 13 *Arabidopsis* accessions** (**A**) Lateral root length (LRL). (**B**) Lateral root number (LRN). Vertical bars on columns indicate standard errors (*n* = 6–8), see legends in Figure 2 for different colors.

Identifying the primary root with certainty was only possible for three accessions (Cvi‐0, Fei‐0, Ts‐1), since the other accessions had several main roots with similar characteristics. Hence, we introduced the term “MRU” as a basic unit for phenotyping RSA (Figure 2; the MRU number (MRUN) is 3, indicated with the ellipses). In this article, the term “main root” will be used for the longest root observed in each MRU, the term “lateral root” (LR) for the first‐order branch roots attached to the main root in individual MRUs and the term “secondary lateral root” (2′‐LR) for the second‐order branch roots attached to “lateral root” in individual MRUs. Given that in most accessions lateral roots were not evenly distributed along the main root, we quantified the distribution of lateral roots in individual MRUs, by dividing them into four sections of equal length, 1Q–4Q (Figure 2) and in each of these categories determined lateral root length (LRL) and number (LRN) (Figure 3).

### Variations of complex RSA

In the quarterly division analysis of lateral root branching, LRL generally showed larger variation than LRN (Figure 3). This was observed in all four quarters and there was a tendency that the variation of these traits increased in the lower sections, where lateral roots have emerged later.

**Figure 4:**
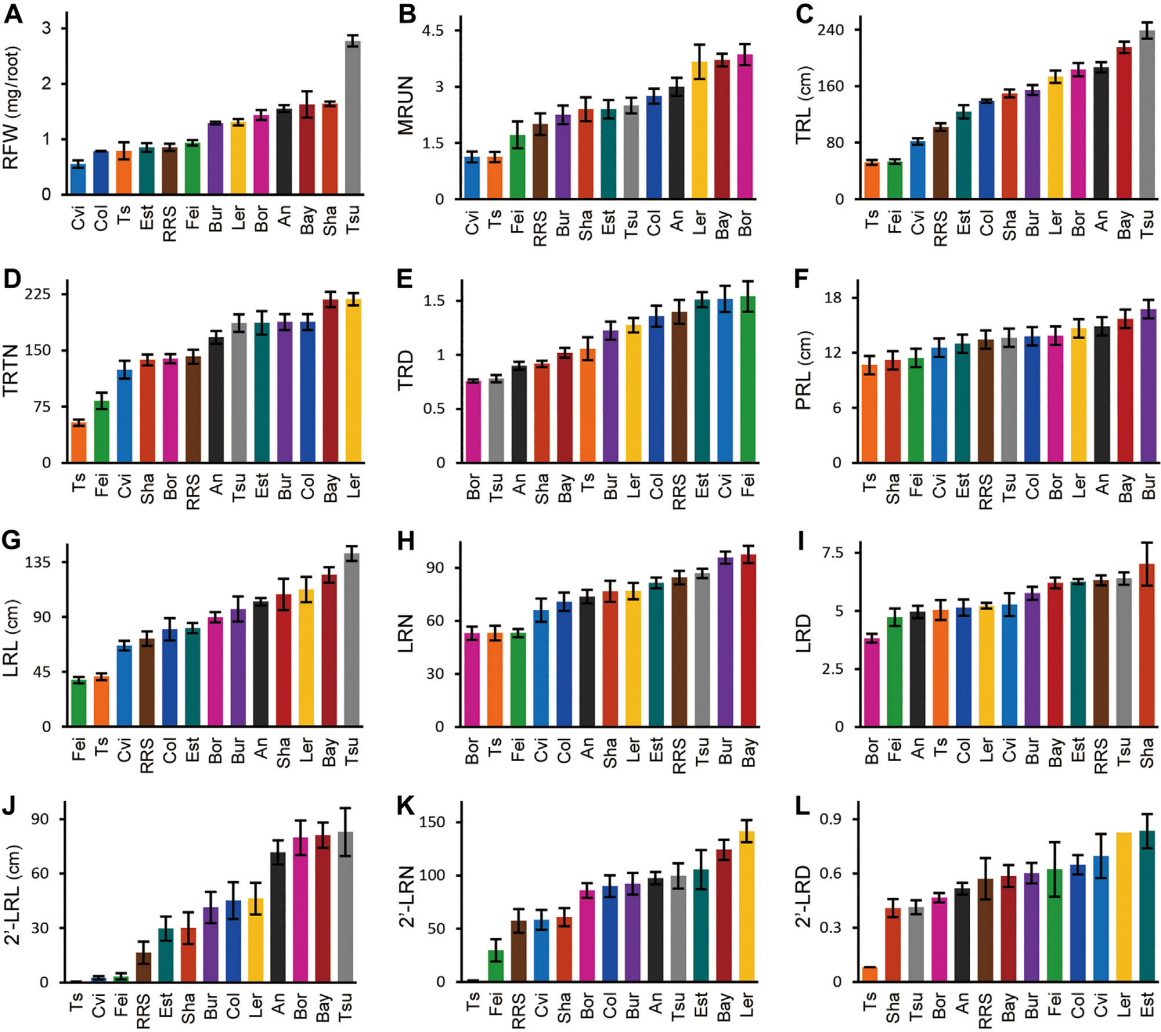
**Natural variations of root system architecture (RSA) of 23‐d‐old roots** (**A**) Root fresh weight (RFW). (**B**) Mature root unit number (MRUN). (**C**) Total root length (TRL). (**D**) Total root‐tip number (TRTN). (**E**) Total root density (TRD = TRTN TRL‐1). (**F**) Primary root length (PRL, as the linear stretch of whole root system). (**G**) Lateral root length (LRL). (**H**) Lateral root number (LRN). (I) Lateral root density (LRD = LRN PRL‐1). (**J**) Secondary‐lateral root length (2′‐LRL). (**K**) Secondary‐lateral root number (2′‐LRN). (**L**) Secondary‐lateral root density (2′‐LRD = 2′‐LRN TRL‐1).

Other root phenotypic traits also varied between accessions, but not all to the same degree (Figure 4). Root fresh weight (RFW) showed more than five‐fold difference between extremes (Cvi, Tsu), ranging from 0.5 to nearly 3 mg. Also mature root unit number (MRUN), total root length (TRL), total root‐tip number (TRTN), LRL and secondary lateral root length (2′‐LRL) displayed large variation. Smaller variation was observed for primary root length (PRL), LRN and lateral root density traits. Just as 2′‐LRL showed larger variation than LRL, also 2′‐LRN displayed a larger variation than LRN. Secondary lateral roots were present in most accessions, except in Ts‐1, which also showed the shortest TRL. Among root traits, primary root length (PRL) showed the smallest variation. Lateral root density (LRD) showed limited variation since it was closely linked to lateral root number, which also showed small variation.

RSA and their hierarchical relationships of 13 accessions in this study were obviously different from those of accessions analyzed in previous studies, in which roots were grown on agar plates (Figure S2) (Armengaud et al. 2009; Kellermeier et al. 2013). Selection of accessions, method of culture and plant age in the present report differ too much from the cited studies to allow direct comparison.

Table 1 shows correlations between root phenotypic traits observed in 23‐d‐old roots of 13 natural accessions. Several obvious correlations were observed, for example between TRL and RFW. Our data allowed the study of more complex traits, including lateral‐root related ones, and we will therefore focus on traits describing the complexity of the root system. RFW was highly correlated with the second quarter of lateral root length (LRL‐2Q, *r* ^2^ = 0.82), followed by LRL‐3Q (*r* ^2^ = 0.7). TRL that is a determinant of RFW was the highest correlated with LRL‐2Q, followed by LRL‐1Q. These correlations imply that LRL‐1Q and LRL‐2Q contribute most to TRL. LRN was the highest correlated with LRL‐3Q, followed by LRL‐2Q, showing that *Arabidopsis* develops more lateral roots in the middle than in the top section of the main root. TRTN, an indicator of branch complexity in the whole root system, was the highest correlated (*r* ^2^ = 0.97) with 2′‐LRN, showing that phenotyping of mature root systems, such as used in the present study, requires sophisticated measurements including secondary and tertiary lateral root traits.

**Table 1:** Correlations between root phenotypic traits

Variables	RFW	MRUN	TRL	TRTN	TRD	PRL	LRL	LRN	LRD	2′‐LRL	2′‐LRN	2′‐LRD	LRL‐1Q	LRL‐2Q	LRL‐3Q	LRL‐4Q	LRN‐1Q	LRN‐2Q	LRN‐3Q
MRUN	0.45																		
TRL	0.82*	0.79*																	
TRTN	0.45	0.71*	0.79*																
TRD	–0.72*	–0.45	–0.68*	–0.15															
PRL	0.29	0.61	0.66	0.80*	–0.17														
LRL	0.83*	0.66	0.96*	0.82*	–0.59	0.58													
LRN	0.40	0.29	0.58	0.79*	–0.05	0.68	0.69*												
LRD	0.31	–0.13	0.21	0.35	0.04	–0.01	0.44	0.73*											
2′‐LRL	0.75*	0.84*	0.95*	0.68	–0.73*	0.65	0.82*	0.40	–0.02										
2′‐LRN	0.42	0.80*	0.80*	0.97*	–0.18	0.77*	0.79*	0.63	0.16	0.72*									
2′‐LRD	–0.20	0.32	0.16	0.64	0.55	0.45	0.20	0.36	0.08	0.09	0.67								
LRL‐1Q	0.66	0.68	0.89*	0.82*	–0.51	0.85*	0.84*	0.65	0.13	0.85*	0.80*	0.27							
LRL‐2Q	0.82*	0.66	0.93*	0.78*	–0.59	0.47	0.98*	0.60	0.41	0.80*	0.76*	0.18	0.74*						
LRL‐3Q	0.70*	0.26	0.59	0.44	–0.41	–0.02	0.76*	0.48	0.71*	0.38	0.38	0.02	0.33	0.80*					
LRL‐4Q	0.24	–0.08	0.04	–0.11	–0.24	–0.42	0.21	0.10	0.60	–0.13	–0.18	–0.23	–0.13	0.20	0.67				
LRN‐1Q	0.23	0.33	0.40	0.56	–0.12	0.75*	0.39	0.71*	0.26	0.37	0.45	0.11	0.64	0.23	–0.05	–0.20			
LRN‐2Q	0.48	0.41	0.62	0.71*	–0.19	0.74*	0.62	0.86*	0.47	0.56	0.58	0.21	0.66	0.53	0.26	–0.12	0.80*		
LRN‐3Q	0.38	0.23	0.55	0.78*	0.03	0.64	0.68	0.97*	0.73*	0.35	0.62	0.44	0.62	0.60	0.50	0.09	0.57	0.76*	
LRN‐4Q	0.08	–0.13	0.13	0.33	0.14	–0.06	0.38	0.49	0.75*	–0.12	0.23	0.31	0.09	0.38	0.67	0.56	–0.11	0.01	0.60

Significant correlations (*P* < 0.01) are marked with an asterisk. RFW, root fresh weight; MRUN, mature root unit number; TRL, total root length, cm; TRTN, total root‐tip number; TRD, total root density; PRL, primary root length as the linear stretch of whole root system; LRL, lateral root length; LRN, lateral root number; LRD, lateral root density; 2′‐LRL, secondary‐lateral root length; 2′‐LRN, secondary‐lateral root number; 2′‐LRD, secondary‐lateral root density.

### Natural variation in hormone concentrations in roots

In total, 33 phytohormones and related metabolites in four different classes were targeted for hormone quantification (Table S1). Eleven phytohormones—auxin, abscisic acid (ABA), gibberellin A_9_ (GA_9_) and eight CKs—could reliably be quantified in 23‐d‐old roots of all 13 accessions. Among 12 targeted GAs, four (GA_1_, GA_3_, GA_7_, GA_20_) were detected in some accessions, whereas GA_9_ was commonly determined in all accessions.

**Figure 5:**
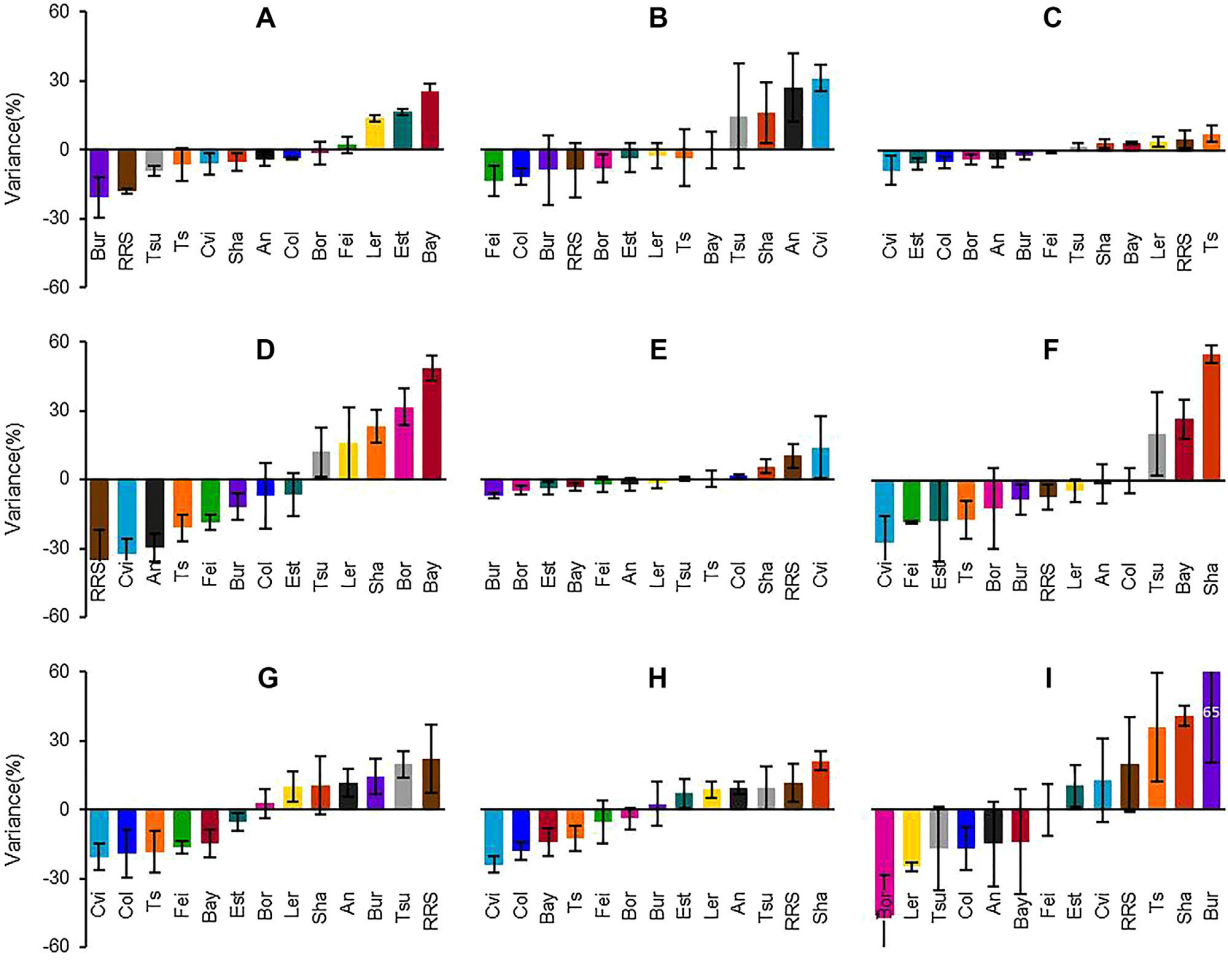
**Natural variations in endogenous hormone levels in 23‐d‐old roots of *Arabidopsis*** (**A**) Indole‐3‐acetic acid (IAA). (**B**) Abscisic acid (ABA). (**C**) Isopentenyl adenine (iP). (**D**) Isopentenyl adenine riboside (iPR). (**E**) *Trans*‐zeatin (tZ). (**F**) *Trans*‐zeatin riboside (tZR). (**G**) *Trans*‐zeatin‐7‐glucoside (tZ7G). (**H**) *Trans*‐zeatin‐O‐glucoside and *trans*‐zeatin‐9‐glucoside (tZ(O, 9)G). (**I**) Gibberellin A_9_ (GA_9_). Y‐axis is percentage of variance, obtained from the formula, Y = ((X‐A)/A)100. X is the hormone concentration in a given accession, and A is the average of concentration of hormone found in all accessions. Vertical bars on columns indicate standard errors (*n* = 3∼4). tZOG and tZ9G were quantified together because peaks of compounds were overlapped in the chromatograms. See Table S2 for absolute quantities of hormones.

Overall, phytohormone levels showed a limited range of variation among accessions (Figure 5). However, the extent of the variation strongly depended on the type of hormone and metabolites. Variations in the levels of auxin and ABA were within ±25% from the average values for all accessions, and the difference between the extremes was less than two‐fold (Table S2). For CKs, the levels of isopentenyl adenine (iP) and *trans*‐zeatin (tZ) were maintained in a very narrow range (±10%), whereas CK ribosides showed relatively larger variations, namely ±50%. *Trans‐*zeatin glucosides (tZG) showed around ±30% variation, larger than for the free bases. The variation in *cis‐*zeatin (cZ) level was within ±50%, similar to the ribosides. GA_9_ displayed larger variation, up to three‐fold, between the extremes.

### Correlations between phytohormone levels and root system architecture

Several significant correlations between phytohormone levels and RSA traits were detected (Table 2). MRUN and TRTN, describing root maturity and global complexity, positively correlated with IAA level, although the correlation was not significant. Secondary lateral root traits also positively correlated with auxin. However, RFW showed little correlation with auxin. These relationships reflect the important role of auxin in shaping the root system, particularly regarding lateral root branching and elongation.

**Table 2:**
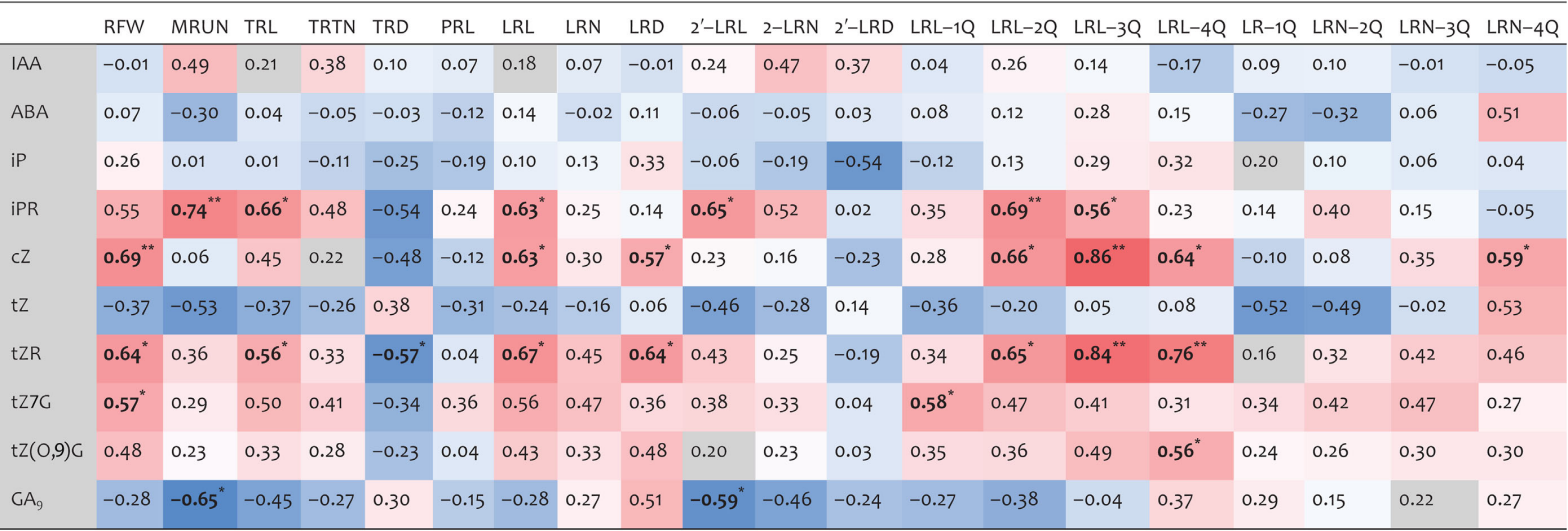
Correlations between hormone levels and RSA

Significant correlations are marked with an asterisk on bold figures: single and double asterisks indicate significance levels at *P* < 0.05 and *P* < 0.01 respectively. Blue and red colors indicate negative and positive correlations respectively. RFW, root fresh weight; MRUN, mature root unit number; TRL, total root length, cm; TRTN, total root‐tip number; TRD, total root density; PRL, primary root length as the linear stretch of whole root system; LRL, lateral root length; LRN, lateral root number; LRD, lateral root density; 2′‐LRL, secondary‐lateral root length; 2′‐LRN, secondary‐lateral root number; 2′‐LRD, secondary‐lateral root density; IAA, indole‐3‐acetic acid; ABA, abscisic acid; iP, isopentenyl adenine; ipR, isopentenyl adenine riboside; cZ, *cis‐*zeatin; tZ, *trans‐*zeatin; tZR, *trans*‐zeatin riboside; tZ7G, *trans‐*zeatin‐7‐glucoside; tZ(O,9)G, *trans*‐zeatin‐O‐glucoside and *trans*‐zeatin‐9‐glucoside; GA_9_, gibberellin A_9_.

Most of the root traits, except TRD and LRN‐4Q, negatively correlated with tZ, for which an antagonistic interaction with IAA in root development has been described (Pernisova et al. 2009; Ruzicka et al. 2009). Another active free base, iP showed different correlations with various root traits, namely RFW, TRD, 2′‐LRD and LRN‐1Q/2Q, indicating that each free base may have different physiological effects on RSA. *Cis‐*zeatin showed significant positive correlations with RFW, LRD, LRL‐2Q/3Q/4Q and LRN‐4Q, and its overall correlation pattern was similar with those of the ribosides and glucosides.

ABA negatively correlated with most of the root traits, except LRN‐4Q, where very short lateral roots (shorter than 0.5 mm) were dominant. Overall correlations of GA_9_ to each root trait were similar with those of tZ, except LRN in the upper main root axis.

**Figure 6:**
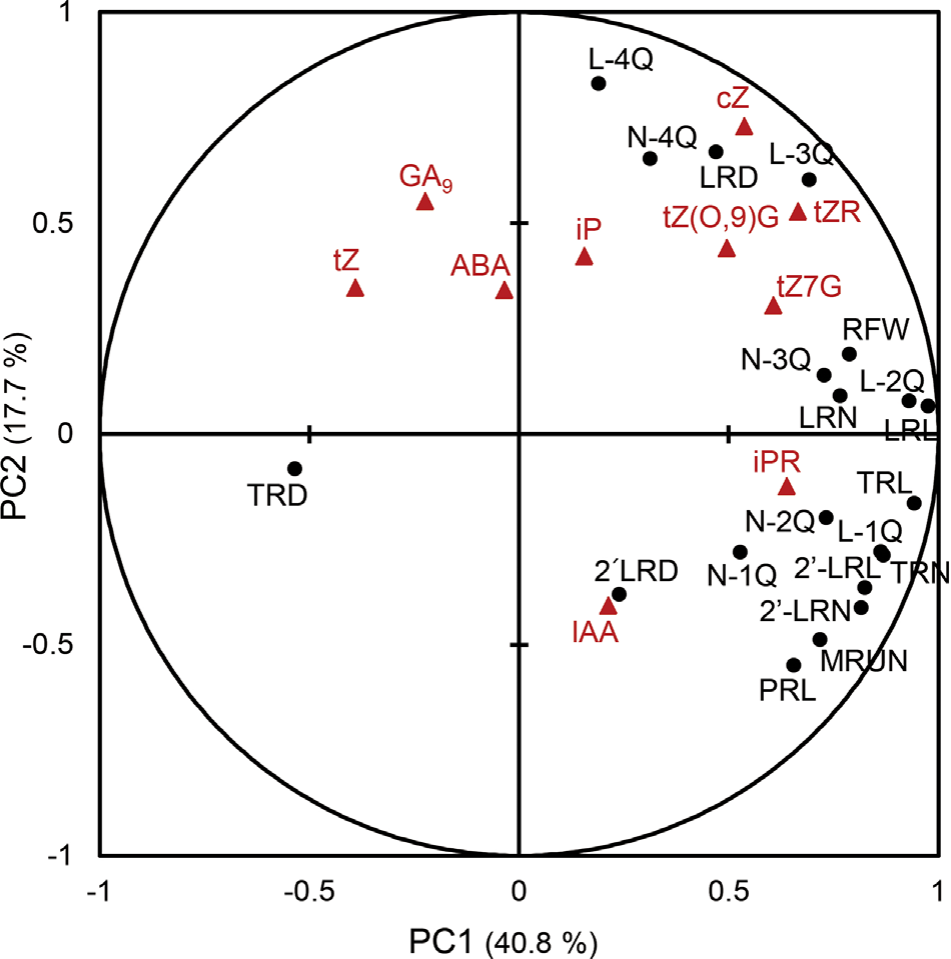
**Principal component analysis of hormone levels and root system architecture (RSA) traits in 23‐d‐old roots of *Arabidopsis*** Red triangles indicate hormone traits, and black circles show root phenotypic trait. Meanings of abbreviations are given in the legends of Figure 4 (root traits) and Figure 5 (hormones). For simplicity, LRL and LRN for quarterly distribution are abbreviated as L‐1Q/2Q/3Q/4Q and N‐1Q/2Q/3Q/4Q, respectively.

In order to have a global view of interactions between phytohormone levels and RSA, we performed a principal component analysis (PCA) based on Pearson's coefficients (Figure 6). Approximately, 58% of the variation was explained by the first two principal components (PC1, PC2), reaching 71% cumulative explanation with PC3 (Table S3). The first principal component (PC1) is composed mainly of all root traits and some CKs, such as tZR, iPR and tZ7G. The other phytohormones mainly contributed to PC2. CK ribosides and glucosides clustered closely with some of the root phenotypic traits; for example, RFW to tZ7G. *Trans‐*zeatin clustered with ABA and GA_9_, which were diagonally opposite to IAA.

### Hormone levels versus hormone sensitivity

**Figure 7:**
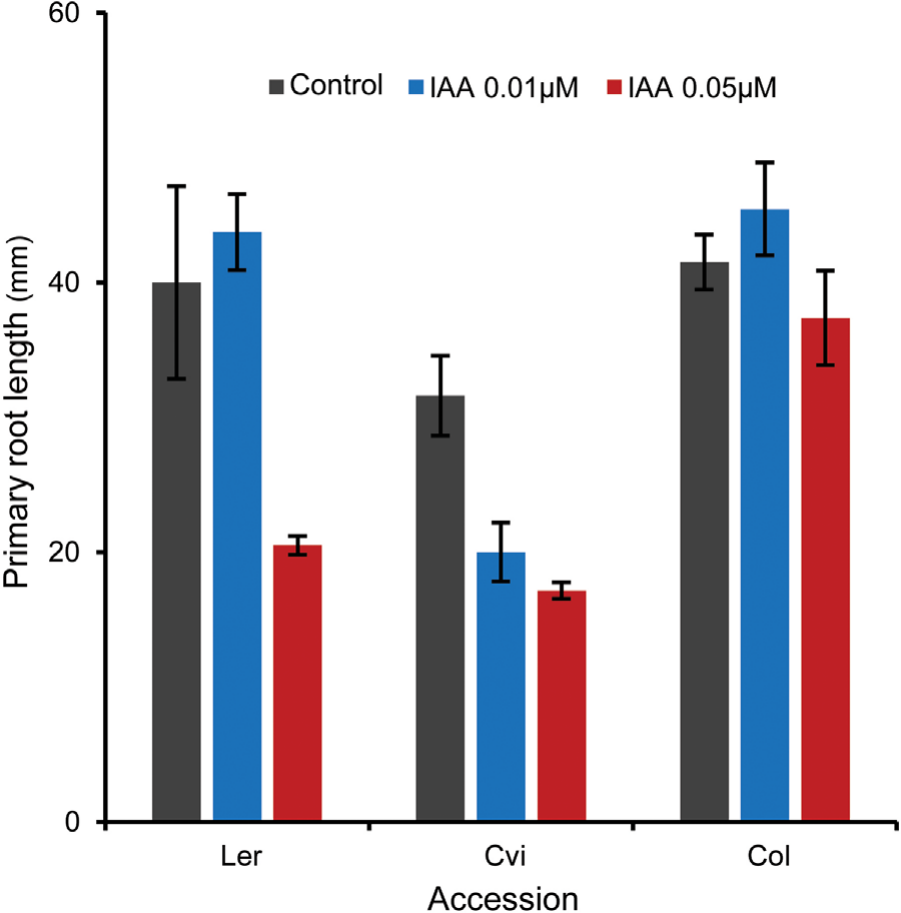
**Effect of indole‐3‐acetic acid (IAA) on primary root length in *Arabidopsis* seedlings** Plants were grown on vertical agar plates with half strength of Murashige and Skoog media, containing IAA. Primary root lengths were measured after 10 d of culturing.

The response of a plant developmental process to hormones is the result of hormone level and the sensitivity toward that hormone. In a pilot experiment we assayed the sensitivity for auxin of three accessions, which have been widely used for the generation of recombinant inbred lines (RILs). Since long‐term application under non‐sterile hydroponic conditions will result in rapid breakdown of IAA, we used a sterile plate‐assay instead. Figure 7 shows that roots of Ler and Col, in the absence of exogenous IAA, are longer than Cvi roots. This is consistent with various root‐length‐related traits as measured for the same accessions in later developmental stages from the hydroponic system (Figure 4). The addition of IAA to the plates resulted in shorter roots. This would imply that IAA inhibits root elongation, a well‐known phenomenon (Rahman et al. 2007; Swarup et al. 2007). Thus, if root elongation would only be controlled by the level of endogenous IAA, levels in Ler and Col would be expected to be lower than in Cvi. This is obviously not consistent with the data presented in Figure 5. Then, can differences in root length between these three accessions be explained by differences in sensitivities toward IAA? Figure 7 shows that Ler, and especially Col are much less sensitive to exogenous IAA than Cvi: within the tested range of concentrations, Col hardly responds, whereas Cvi shows a 30% decrease in root length already at the lowest concentration tested. Ler showed an intermediate behavior.

### Quantitative changes in hormone contents during mature root development

Quantitative changes and profiling of phytohormones in organs/tissues have been reported in some plants (Taylor and vanStaden 1997; Kanno et al. 2010; Powell et al. 2013). In *Arabidopsis*, most studies on hormone quantification have focused on young seedlings, and hence, data on dynamic changes during rapid root growth in later stages are missing. Information on quantitative changes in hormones, if occurring, may help to understand their biochemical and functional roles in root growth. Therefore, we measured quantitative changes in phytohormones in the roots of two representative accessions (Col‐0, Ler‐0), which showed vigorous vegetative growth during five weeks (23–58 d, before transition to flowering) (Figure S3).

**Figure 8:**
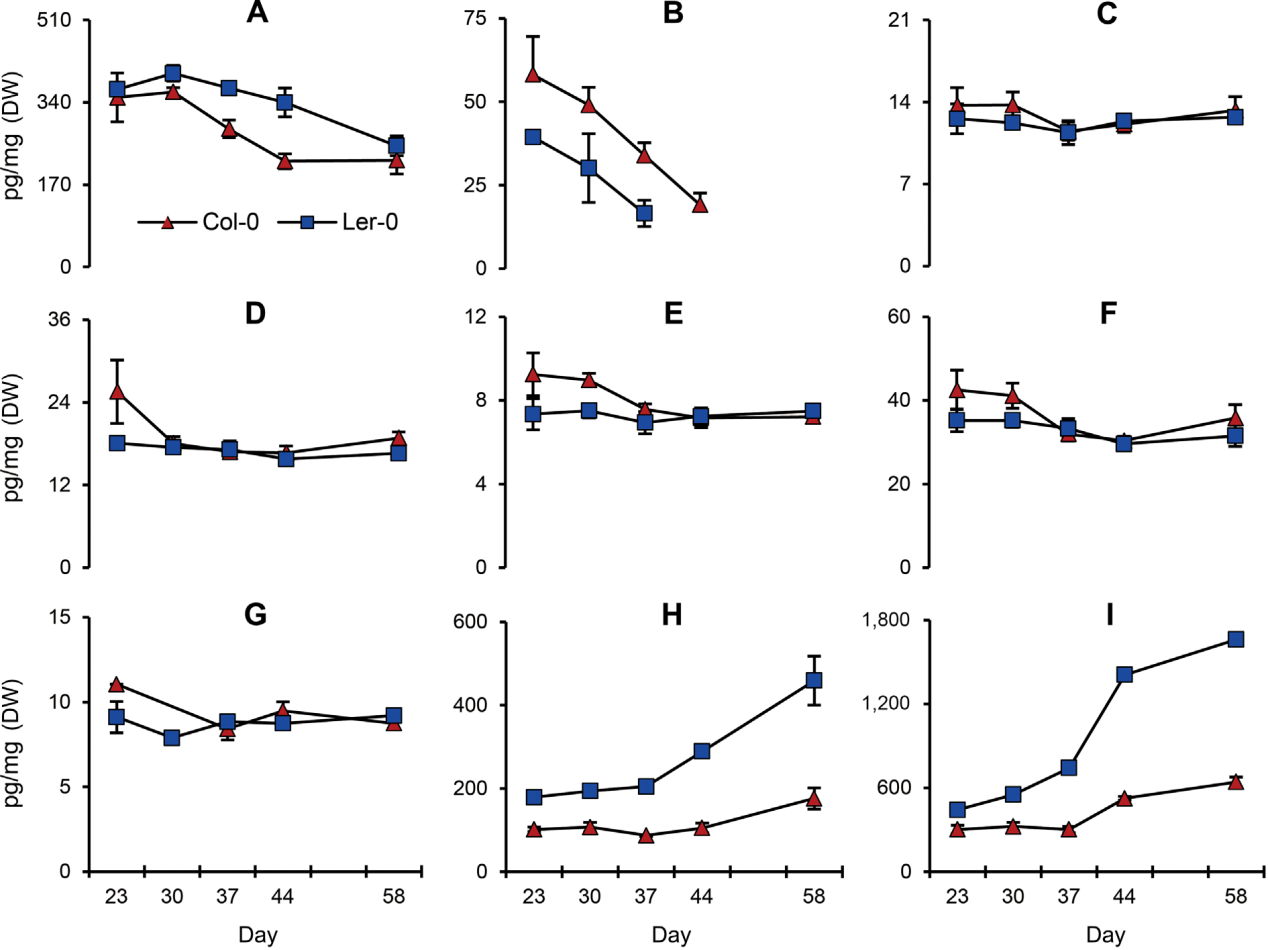
**Temporal changes of hormone levels in *Arabidopsis* roots during mature root development** (**A**) Indole‐3‐acetic acid (IAA). (**B**) Abscisic acid (ABA). (**C**) Isopentenyl adenine (iP). (**D**) Isopentenyl adenine riboside (iPR). (**E**) *Trans‐*zeatin (tZ). (**F**) *Trans*‐zeatin riboside (tZR). (**G**) Dihydrozeatin riboside (DZR). (**H**) *Trans‐*zeatin‐7‐glucoside (tZ7G). (**I**) *Trans*‐zeatin‐O‐glucoside and *trans*‐zeatin‐9‐glucoside (tZ(O,9)G). Red triangles and blue squares indicate Columbia‐0 and Ler‐0 respectively. Unit of Y‐axes is pg/mg in dry weight commonly. Vertical bars on markers indicate standard errors (*n* = 4). Data of 51st d were missed. ABA levels were below the detection limit after 37 d for Ler‐0 and 44 d for Col‐0.

Overall, each phytohormone showed similar time‐course patterns in both accessions (Figure 8), although there were some minor differences. Auxin level decreased between 30 and 58 d in both accessions, with a 40% decrease in Col‐0 and 32% in Ler. ABA sharply decreased in both accessions from 23 d to around 40 d. Thereafter, ABA levels were below the detection limit. Levels of CK free bases were fairly stable for the whole period, although tZ in Col‐0 slightly decreased (22%) between 23 and 44 d. CK ribosides (iPR, tZR, DZR) did not change much in both lines, while levels of glucosides (tZ7G, tZ(O,9)G) gradually increased after 37 d: tZ7G, 73% in Col‐0 and 156% in Ler; tZ(O,9)G, 112% in Col‐0 and 274% in Ler. Levels of dihydrozeatin (DZ) and GAs, which could be measured in 23‐d‐old roots, were below the detection limit after 30 d.

## DISCUSSION

Roots are vital for plant growth and survival, and their development should adequately respond to environmental cues. Hormones are supposed to be essential in this process, transducing the external signals to internal ones. Different accessions of one species may have adapted to a specific local environment, thus resulting in natural variation within the species for root‐related traits. We tried to answer the question whether, and to what extent, endogenous hormones are involved in such adaptations, by determining endogenous levels of a series of hormones, and how they correlate with root phenotypic traits. Since the root architecture in later stages of the development plays an important role in the adaptation to the environment, we decided to focus on mature plants, rather than seedlings that have been investigated in many other studies related to root architecture (Zhu et al. 2011; Slovak et al. 2014).

### Evaluation of mature RSA requires proper root phenotypic traits

Variation in phenotypic traits in 23‐d‐old mature roots was large and different for each trait, similar as found in growth‐related traits in the shoot (El‐Lithy et al. 2004). The larger variation in LRL traits, as compared to LRN, for example, shows that *Arabidopsis* displays a smaller genetic variation for lateral root numbers than for length. The obvious differences in lateral root lengths, despite the homogeneous hydroponic culture, suggest phenotypic plasticity of *Arabidopsis* root system to environmental constraints, for example drought, may be considerable. A large variation was also observed in root weight, as was shown earlier for young seedlings (Clark et al. 2013; Slovak et al. 2014). However, PRL and LRD, important traits to evaluate phenotypes of seedling roots, were less variable in the present study, suggesting that phenotypic traits of interest in mature roots should be carefully chosen for proper description and quantification.

### *Arabidopsis* roots show homeostasis for phytohormone levels

The natural variation in the levels of hormones in the present study was surprisingly low, differences being less than two‐fold between extremes, except for GA_9_. These conserved levels of hormones are in stark contrast with other studies on primary and secondary metabolites in plants. The concentrations of flavonoids in seeds of 41 *Arabidopsis* ecotypes showed large variation: 39‐fold for quercetin‐3‐rhamnoside and 9.6‐fold for biflavonols (Routaboul et al. 2012). There was a 20‐fold difference for total aliphatic glucosinolate levels among leaves of 39 *Arabidopsis* ecotypes (Kliebenstein et al. 2001). Also in a metabolite study in tomato, the variation in levels of primary and secondary metabolites was large, for instance, tocopherol contents showed 10‐fold differences between extreme lines (Sauvage et al. 2014).

Earlier reports described large natural variation in the response to hormones and stresses, as detected in transcriptome studies (Delker et al. 2010; Sofo et al. 2013). The limited variation in hormone levels, including biologically inactive conjugates and other products of inactivation that we found in the present study, would imply that levels of phytohormones are controlled by sophisticated homeostasis mechanisms, presumably involving conjugation, oxidation, transport and synthesis. More fine‐tuned homeostatic control can be found for active hormone compounds, for example CK‐free bases, as described in the present study, which are regulated in coordination with complex signaling mechanisms in non‐linear pathways (Vanstraelen and Benkova 2012).

It should be noted, however, that hormone levels in our study were determined in a whole‐root extract, thus not including information on possible differential concentrations, which have been shown at the cellular or tissue level (Bielach et al. 2012; Antoniadi et al. 2015). Although incomplete, the present data are still valuable to advance our understanding of the natural variation in homeostatic regulation of phytohormones between active forms and intermediates in *Arabidopsis* roots.

The variation in auxin level (±25%) in our study can be a consequence of two features: genetic polymorphism for genes involved in auxin metabolism, and variation for transporters, such as PINs/ABCBs and AUXs (Kramer and Bennett 2006; Petersson et al. 2009), resulting in local differences of levels in the root system. The larger variation in the level of GA_9_ may be a feature of an inactive GA as a precursor for the biologically active GA_4_ (Hedden and Thomas 2012), but more quantitative information for other GA species is required to understand the natural variation for this large class of hormones, which are relatively more abundant in leaves than in roots (Nam et al. 2017).

### Correlation analysis between hormones and RSA may help to understand the hormonal cross‐talk that determines root architecture

Positive correlations of auxin level to TRTN and TRL were consistent with previous findings in seedlings. This would imply that the local maxima for auxin in the root apices, as observed in seedlings, are also present in mature roots. In our study, RSA seems to be controlled mainly by antagonistic relationships between IAA on the one hand, and CKs (tZ, iP), ABA and GA_9_ on the other hand (Figure 6). Cross‐talks between CK signaling and *PIN‐FORMED* (*PIN*) expression occur in early embryonic root development (Muller and Sheen 2008; Bishopp et al. 2011). The underlying mechanism may be that CK suppresses the expression of *PIN1*, thus depleting polarly localized *PIN* proteins at the basal membrane, consequently decreasing directional auxin flow (Marhavy et al. 2014). Auxin‐CK balance also regulates the root meristem size in *Arabidopsis*, through triggering of CK signaling, thus affecting root biomass (Dello Ioio et al. 2008; Ruzicka et al. 2009).

Another antagonistic hormone interaction for mature root development that showed up in the PCA was that between GA_9_ and auxin (Figure 6). According to Moubayidin et al. (2010), GA_3_ induces *PIN* expression via repression of *ARABIDOPSIS RESPONSE REGULATOR 1* (*ARR1*) and *IAA3/SHY2* transcription but attenuates CK activity. This positive regulation of auxin by GA conflicts with the present study that GA_9_ clustered with tZ rather than with IAA. This discrepancy may be due to the large differences between various GAs, with the biologically active GAs (GA_3_, GA_4_), showing opposite behavior from the inactive ones, such as GA_9_, similar to the opposite behavior between CK ribosides (iPR, tZR) and tZ as seen in the PCA (Figure 6).

In the present study, clustering of ABA with tZ could be compatible with the results presented by Shkolnik‐Inbar and Bar‐Zvi (2010), showing that LR growth can be altered by the *ABSCISIC ACID INSENSITIVE 4* (*ABI4*) transcription factor, the expression of which is enhanced by ABA and CK, and reduces polar auxin transport, finally causing inhibition of LR formation. Overall, these possible hormonal cross‐talks seem also compatible with previous results, showing comparable phenotypic plasticity in wild‐type (Col‐0) and a number of *Arabidopsis* mutants (*axr4*, *abi4*, *cre1*) under various treatments (IAA, ABA, CK) (Ristova et al. 2013).

The experiment with exogenously applied hormone (Figure 7) shows the importance of hormone sensitivity in controlling root growth. However, the various significant positive and negative correlations between hormone levels and root traits (Table 2) also indicate a prominent role for the endogenous hormone levels themselves.

### Changes in hormone concentrations during root growth may reflect changes in spatial distribution and localization

Based on quantitative changes during root development (Figure 8), phytohormones can be divided into three groups: decreasing (IAA, ABA), constant (tZ, tZR, iP, iPR) and increasing (tZ7G, tZ(O,9)G). Auxin levels show local maxima in root apices (Grieneisen et al. 2007, Petersson et al. 2009); thus the gradual decrease in the auxin level during root development and elongation likely reflects a dilution effect, with relatively fewer apices as compared to total root mass in older roots. Hydroponic culture provides a homogenous environment for roots that may normally be sensitive to dryness in the rhizosphere, inducing local ABA accumulation as observed in potatoes (Puertolas et al. 2015). In maize, younger roots have higher ABA levels than older roots (Zhang and Tardieu 1996), which is consistent with the decrease in ABA levels during root maturation in the present study.

In a current model of CK biosynthesis in plants, ribosides and free bases are synthesized through two paths: one is from tZRMP to tZR and finally to tZ, including two enzymatic steps and the other is from tZRMP directly to tZ catalyzed by LONELY GUY (LOG) without tZR as an intermediate (Kamada‐Nobusada and Sakakibara 2009). Studies using multiple mutants and overexpression lines for *LOG* genes, show that the levels of CK ribosides and free bases are cumulatively affected by LOG homologues, each of them having a small additive effect (Kuroha et al. 2009, Tokunaga et al. 2012). Both non‐linear biosynthetic pathways and polygenic regulation for a given enzymatic conversion may support long‐term homeostasis of ribosides and free bases, as revealed in the present study of *Arabidopsis* roots.

CK *N‐*glucosides are products of a linear metabolic pathway and are synthesized irreversibly from free bases by N‐glucosyltransferases, followed by CK oxidases/dehydrogenases (*CKX*) for degradation. The significant positive correlation of tZ7G with RFW and TRL in the 23‐d‐old roots and the accumulation of tZ(7,9)G during rapid root growth are consistent with the findings of Kollmer et al. (2014) that decreased levels of *N‐*glucosides, due to overexpression of *CKX*7, result in an early termination of the primary root growth, complete suppression of lateral root initiation and aberration of root vascular development. It is yet unknown how accumulation of CK *N‐*glucosides is controlled, their levels depending on two enzymatic reactions, between *N‐*glucosylation and oxidation. Presumably, CK *N‐*glucosides may have a physiological role in *Arabidopsis* root growth, different from *O‐*glucosides that are relevant for homeostatic regulation of biologically active CK.

In summary, here we report that endogenous hormone levels in roots of natural accessions of *Arabidopsis* are maintained within a narrow range of concentrations. Complex RSA in mature plants, consisting of multiple‐order lateral roots, should be studied using appropriate phenotypic traits, since the use of only two components—the primary and the lateral roots—structuring young roots, is not suitable to effectively describe mature root systems. In correlation analyses we showed that some phenotypic traits in mature roots can be explained by hormone cross‐talk. Since natural variation is an important premise to unravel genetic elements through quantitative traits analyses (Koornneef et al. 2004), this study suggests that quantitative trait loci analysis for phytohormone levels is feasible, using mapping populations derived from divergent accessions, for example, Ler and Cvi (Figure S4). This way the molecular mechanisms, by which root architecture in plants is determined, will be further unraveled.

## MATERIALS AND METHODS

### Plant growth and sampling

Thirteen *Arabidopsis thaliana* L. Heynh. accessions (An‐1, Bayeuth‐0, Bor‐4, Bur‐0, Columbia‐0, Cvi‐0, Est‐1, Fei‐0, Ler‐0, RRS‐7, Shahdara‐0, Ts‐1, Tsu‐0) from the *Arabidopsis* Seed Stock of the Laboratory of Genetics at Wageningen University were used. For germination, seeds were placed on wet filter paper in a Petri dish at 4°C in darkness for 4 d, seeds were then sowed on the top of 0.5 mL tubes, with the bottom cut off, and filled with 0.5% agar in half strength Hoagland's nutrient solution (pH 5.5). Tubes with germinating seeds were placed in a hydroponics tank filled with half strength of Hoagland's nutrient solution (pH 5.5, 9 L), renewing the solution once a week and approximately 70 plants per tank). Plants were kept at 21°C during the light period (10 h) and at 18°C during the dark period (14 h), from sowing (day 0) until harvesting. Light intensity and humidity were fixed at 125 µmol photons m^−2^ per second and 70%, respectively.

To avoid that individual root systems get entangled wsith roots from neighboring plants, each whole root system was comparted individually in a plastic column (diameter 3 cm, depth 6 cm, open at top and bottom) in the hydroponics tank (Figure S1). After 23 d of culture, roots were harvested between the 5^th^ and 8^th^ h during the 10 h light period for hormone analysis and phenotyping. Root length and weight were measured. Thereafter, roots of five to six plants were pooled for a biological replicate. Four biological replicates of each accession were used for hormone analyses. Pooled roots were ground in liquid nitrogen and freeze‐dried for 24 h. Ten intact roots of each accession, which were grown at the same batch as for hormone analyses, were also harvested, put into Petri dishes filled with water and preserved at −20°C until two‐dimensional phenotyping.

### Two‐dimensional root image and phenotyping

Frozen roots were defrosted in two steps to prevent roots from being broken into pieces: at 4°C for 2 h and after that at room temperature (∼22°C). Roots were spread in square Petri dishes (12.5 × 12.5 cm), containing 0.1% Tween20 in water. Each branch root was unraveled from other roots with a brush in such a way that the whole root system was kept intact without any overlapping among roots. Two‐dimensional root images were generated by a photo scanner (Epson, Perfection V700). The software package, WinRhizo (Regent Instruments Inc., Canada), was used to measure total root length (TRL, cm) from the resulting TIF file image. Total root‐tip number (TRTN) was counted based on all emerged roots that were longer than 0.5 mm in length. In most cases identifying the primary root was not possible, since lateral roots had formed, which were indistinguishable from the primary root in length and diameter. Therefore, we introduced a new descriptor, as a basic unit of phenotyping, “mature root unit (MRU)” to measure complex root architecture (Figure 2). Lateral root number (LRN) and lateral root length (LRL) were assessed separately for each MRU. Within each MRU, lateral roots were further divided into four sections of equal length from top to bottom, and numbers (N) and lengths (L) were determined, resulting in these parameters: LRN‐1Q/2Q/3Q/4Q and LRL‐1Q/2Q/3Q/4Q. Traits of the secondary lateral roots attached to the lateral roots were designated as 2′‐LRN and 2′‐LRL. To measure real length of roots in scanned images, the ruler dimension of ImageJ was corrected based on dimensions of the Petri dish. After blotting the roots with soft paper tissues, root fresh weight (RFW, mg/root) was determined in three to four replicates, each replicate consisting of roots from five to six plants. For the experiment on the dynamics of hormone concentrations during development, also root dry weight (RDW, mg/root) was measured after 24 h of freeze‐drying.

### Sensitivity test of primary root growth toward exogenous IAA

For germination, seeds (Ler‐0, Col‐0, Cvi‐0) were placed on wet filter paper in a Petri dish at 4°C in darkness for 4 d, germinated seeds were then sowed on vertical plates with half strength of Murashige and Skoog media with 0.8% agar and 1% sucrose, containing IAA (0.01 μmol/L, or 0.05 μmol/L). Plants were kept at 23°C during the light period (16 h) and at 20°C during the dark period (8 h), from sowing (day 0) until harvesting. Light intensity and humidity were fixed at 125 μmol photons m^−2^ per second and 70%, respectively. Primary root lengths were measured after 10 d.

### Hormone extraction and purification

For each accession, four biological replicates were used to extract and quantify endogenous hormones. Powder of lyophilized roots (2.5 mg), as a replicate, was sonicated with 2 mL of methanol: water: formic acid (15:4:1, v/v/v), containing isotope‐labelled internal standards (each chemical, 100 nmol/L final concentration, OlChemIm Ltd, Czech Republic, see supplementary data, Table S1) for 15 min and extracted by shaking for 2 h at 4°C in darkness. After centrifugation at 1 200 g for 10 min (swinging bucket rotor‐type, Model‐The Centaur2, MSE, UK), the supernatant was collected, and the pellet was re‐extracted with 2 mL of ethylacetate: formic acid (19:1, v/v) for 2 h at 4°C in darkness. Pooled supernatants were evaporated to dryness under vacuum (SpeedVac Concentrator, Savant SC210A and refrigerated vapour trap, Savant RVT5105, Thermo, US). The extract was suspended in 1 mL of methanol: water: formic acid (15:4:1, v/v/v) and was purified on an Oasis‐HLB column (150 mg, Waters, US) using the following procedure: the column was activated with 6 mL of methanol, followed by 4 mL of water. After loading the sample, the column was washed with 1 mL of water. Phytohormones were eluted with two sequential eluents: 1 mL of methanol: formic acid (99.9:0.1, v/v) and 2 mL of methanol: formic acid (99:1, v/v). Both fractions were combined and dried under vacuum.

### Quantitative analysis of phytohormones

Quantitative analysis of hormones was conducted using an Acquity UPLC® System (Waters, US) coupled with a triple quadrupole mass spectrometer (Xevo™ TQ, Waters). Purified samples were suspended in 200 μL of acetonitrile: water: formic acid (5:95:0.1, v/v/v), filtered through 0.45 μm polytetrafluoroethylene membrane (Phenomenex, US) and injected onto an Acquity UPLC BEH C_18_ column (100 × 2.1 mm, 1.7 μm; Waters). Two independent injections were chromatographed with different mobile phase schemes to separate targeted compounds: one for IAA, ABA and GAs, the second for CKs. For IAA, ABA and gibberellic acids, 20 μL of sample were injected and eluted by a binary gradient, consisting of 0.1% formic acid in water (A) and 0.1% formic acid in acetonitrile (B), for 11 min at constant flow rate (0.5 mL/min) at 50°C of analytical column temperature for 11 min. The linear gradient elution was performed as follows: 0–1.0 min, 5% eluent B; 1.0–6.67 min, 5%–50% eluent B; 6.67–7.33 min, 50%–90% eluent B; 7.33–9.0 min, 90% eluent B; 9.0–9.5 min, 90%–5% eluent B. At the end of gradient, the column was equilibrated to initial conditions for 1.5 min. For cytokinins, 20 μL of sample was eluted by another mobile phase gradient with the same A and B mobile phases at constant flow rate (0.6 mL/min) at 50°C of analytical column temperature for 14 min. The linear gradient elution was performed as follows: 0–1.5 min, 0.2% eluent B; 1.5–8.5 min, 0.2%–20% eluent B; 8.5–9.5 min, 20%–70% eluent B; 9.5–10.2 min, 70% eluent B; 10.2–10.5 min, 70%–0.2%. At the end of gradient, the column was equilibrated to initial conditions for 3.5 min. The effluent was introduced in electrospray ionization source (ESI) of mass spectrometer with operating parameters: capillary voltage, 3 kV; cone voltage, 22 V; source and desolvation temperature, 150°C and 650°C; cone and desolvation gas flow, 50 and 1,000 L/h; mass spectrometry (MS) mode collision energy, 2 V; MS/MS mode collision energy, 10 V. Two selective transitions were used to perform multiple reaction monitoring (MRM) detections (Table S1). MRM for each compound was grouped into a few distinct functions in order to reduce loss‐of‐signal during monitoring times. Data were processed by TargetLynx in MassLynx™ Software (Version 4.1, Waters). The quantification of each targeted analyte was determined using a linear calibration curve that covered the range of concentrations of compounds in samples and corrected by the recovery rate of the deuterium‐labelled internal standard (^14^C for IAA).

### Statistical analysis

Correlations were calculated using XLSTAT (http://www.xlstat.com). A linear model of Pearson's coefficient was used for PCA. Hierarchical cluster analysis was performed in R program using hclust package https://CRAN.R-project.org.

## AUTHOR CONTRIBUTIONS

S.L. performed most of the research and wrote the manuscript, L.S. co‐supervised the study, carried out the IAA sensitivity test, and revised the manuscript and D.V. supervised the study and revised the manuscript.

## Supporting information

Additional Supporting Information may be found online in the supporting information tab for this article: http://onlinelibrary.wiley.com/doi/10.1111/jipb.12617/suppinfo

**Figure S1.** Illustration of hydroponic cultureThe black plate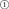 is placed and covered on the hydroponic tank. A sprout on the top of holder‐tube (0.5 mL)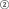 filled with 0.5% agar in half of Hoagland's nutrient solution, grows and develops root system in the hydroponic tank. A small ring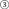 inside the tube was tightly placed to prevent agar medium from slipping down out of the tube during culture, with no physical hindrance for root development. In order to avoid roots being entangled with roots of neighbor plants, a polypropylene column (diameter 3 cm height 5.5 cm)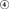 was equipped underneath the black plate, allowing nutrient solution and roots not to be blocked on the bottom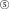. Right picture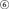 shows how upper shoots settle and grow on the black plate.
**Figure S2.** Hierarchical cluster analysis of RSA traits in 13 *Arabidopsis* accessions
**Figure S3.** Dry root weights of Col‐0 and Ler‐0 during 5 weeks of root developmentData of 51^th^ day was missed.
**Figure S4.** Re‐partition of 13 *Arabidopsis* accession in PCA of hormone levels and RSA traits
**Table S1.** Summary of multiple reaction monitor (MRM) transitions used for hormone quantification in ESI‐TQ mass spectrometer
**Table S2.** Hormone levels in 23‐d‐old roots of 13 *Arabidopsis* accessions (unit: pg/mg dry weight)
**Table S3.** Contribution of variables (hormones and root phenotypic traits) on PCA (unit: %)
