# Supplementary material for: Natural variation of hormone levels in Arabidopsis roots and correlations with complex root architecture
Source: J Integr Plant Biol. 2018 Feb 6;60(4):292–309. doi: 10.1111/jipb.12617 (PMC5947113; doi:10.1111/jipb.12617)
Supplement: Supplementary file 1 — Figure S1. Illustration of hydroponic culture The black plate is placed and covered on the hydroponic tank. A sprout on the top of holder‐tube (0.5 mL) filled with 0.5% agar in half of Hoagland's nutrient solution, grows and develops root system in the hydroponic tank. A small ring inside the tube was tightly placed to prevent agar medium from slipping down out of the tube during culture, with no physical hindrance for root development. In order to avoid roots being entangled with roots of neighbor plants, a polypropylene column (diameter 3 cm height 5.5 cm) was equipped underneath the black plate, allowing nutrient solution and roots not to be blocked on the bottom. Right picture shows how upper shoots settle and grow on the black plate. Figure S2. Hierarchical cluster analysis of RSA traits in 13 Arabidopsis accessions Figure S3. Dry root weights of Col‐0 and Ler‐0 during 5 weeks of root development Data of 51th day was missed. Figure S4. Re‐partition of 13 Arabidopsis accession in PCA of hormone levels and RSA traits Table S1. Summary of multiple reaction monitor (MRM) transitions used for hormone quantification in ESI‐TQ mass spectrometer Table S2. Hormone levels in 23‐d‐old roots of 13 Arabidopsis accessions (unit: pg/mg dry weight) Table S3. Contribution of variables (hormones and root phenotypic traits) on PCA (unit: %) [file JIPB-60-292-s001.docx]

**SUPPORTING INFORMATION**

**Table S1. Summary of multiple reaction monitor (MRM) transitions used for hormone quantification in ESI-TQ mass spectrometer**

| Analytes | UPLC  Gradient | RT | ESI | MRM  Function | Transition (m/z) | Cone voltage | Collision energy | Internal standard | RT | Transition  (m/z) | Cone voltage | Collision energy |
| --- | --- | --- | --- | --- | --- | --- | --- | --- | --- | --- | --- | --- |
| tZ | 1 | 3.78 | + | 2 | 220.1>136.1 | 20 | 18 | [^2^H_6_] tZ | 3.75 | 225.1>136.9 | 20 | 18 |
| tZR | 1 | 5.04 | + | 3 | 352.1>136.1 | 28 | 20 | [^2^H_5_] tZR | 5.01 | 357.1>136.9 | 28 | 20 |
| iP | 1 | 6.15 | + | 1 | 204.1>136.1 | 20 | 18 | [^2^H_6_] iP | 6.11 | 210.1>137.1 | 20 | 18 |
| iPR | 1 | 7.60 | + | 1 | 336.1>204.1 | 28 | 18 | [^2^H_6_] iPR | 7.55 | 342.2>210.2 | 28 | 18 |
| cZ | 1 | 4.01 | + | 2 | 220.1>136.1 | 20 | 18 | [^2^H_6_] tZ | 3.75 | 225.1>136.9 | 20 | 18 |
| cZR | 1 | 5.31 | + | 3 | 352.1>136.1 | 28 | 16 | [^2^H_5_] tZR | 5.01 | 357.1>136.9 | 28 | 20 |
| tZOG | 1 | 3.84 | + | 2 | 382.1>220.1 | 30 | 16 | [^2^H_5_] tZ_9_G | 4.02 | 387.2>225.2 | 30 | 20 |
| tZ_7_G | 1 | 3.86 | + | 2 | 382.1>220.1 | 30 | 20 | [^2^H_5_] tZ_9_G | 4.02 | 387.2>225.2 | 30 | 20 |
| tZ_9_G | 1 | 4.04 | + | 2 | 382.1>220.1 | 30 | 20 | [^2^H_5_] tZ_9_G | 4.02 | 387.2>225.2 | 30 | 20 |
| DZ | 1 | 3.95 | + | 2 | 222.1>136.1 | 20 | 18 | [^2^H_3_] DZ | 3.91 | 225.1>136.1 | 20 | 18 |
| DZR | 1 | 5.10 | + | 3 | 354.1>136.1 | 28 | 22 | [^2^H_3_] DZR | 5.06 | 357.2>149.1 | 28 | 22 |
| DZ_7_G | 1 | 4.07 | + | 2 | 384.1>136.1 | 30 | 20 |  |  |  |  |  |
| DZ_9_G | 1 | 4.17 | + | 2 | 384.1>136.1 | 30 | 20 |  |  |  |  |  |
| DZROG | 1 | 5.12 | + | 3 | 516.1>222.1 | 34 | 20 |  |  |  |  |  |
| tZRMP | 1 | 3.58 | + | 2 | 432.1>220.1 | 34 | 22 |  |  |  |  |  |
| tZROG | 1 | 4.94 | + | 3 | 514.1>382.2 | 34 | 22 |  |  |  |  |  |
| mT | 1 | 4.92 | + | 3 | 242.1>77.0 | 20 | 18 |  |  |  |  |  |
| mTR | 1 | 6.45 | + | 1 | 374.1>242.1 | 30 | 20 |  |  |  |  |  |
| oT | 1 | 5.94 | + | 1 | 242.1>136.1 | 20 | 18 |  |  |  |  |  |
| oTR | 1 | 7.55 | + | 1 | 374.1>242.1 | 28 | 20 |  |  |  |  |  |
| IAA | 2 | 4.15 | + | 1 | 176.1>130.1 | 18 | 16 | [^13^C_6_] IAA | 4.14 | 182.1>136.1 | 18 | 16 |
| ABA | 2 | 4.80 | - | 5 | 263.1>219.1 | 18 | 14 | [^2^H_6_] ABA | 4.77 | 269.1>225.1 | 18 | 14 |
| GA_1_ | 2 | 3.67 | + | 4 | 349.2>285.2 | 20 | 16 | [^2^H_2_] GA_1_ | 3.65 | 351.2>287.2 | 20 | 16 |
| GA_3_ | 2 | 3.61 | - | 7 | 345.2>239.2 | 28 | 14 | [^2^H_2_] GA_3_ | 3.60 | 347.2>241.2 | 28 | 14 |
| GA_4_ | 2 | 6.15 | - | 6 | 331.2>257.2 | 28 | 14 | [^2^H_2_] GA_4_ | 6.13 | 333.2>259.2 | 28 | 14 |
| GA_5_ | 2 | 4.83 | + | 3 | 331.2>285.2 | 12 | 10 |  |  |  |  |  |
| GA_7_ | 2 | 6.06 | - | 6 | 329.2>223.2 | 26 | 14 | [^2^H_2_] GA_7_ | 6.04 | 331.2>225.2 | 26 | 14 |
| GA_8_ | 2 | 2.69 | + | 4 | 365.2>301.2 | 14 | 10 |  |  |  |  |  |
| GA_9_ | 2 | 7.18 | + | 2 | 317.2>271.2 | 16 | 14 | [^2^H_2_] GA_4_ | 6.13 | 333.2>259.2 | 28 | 14 |
| GA_19_ | 2 | 4.80 | + | 3 | 363.2>299.2 | 14 | 12 |  |  |  |  |  |
| GA_20_ | 2 | 4.95 | + | 3 | 333.2>287.2 | 18 | 12 | [^2^H_2_] GA_20_ | 4.93 | 335.2>289.2 | 18 | 12 |
| GA_44_ | 2 | 5.05 | + | 3 | 347.2>301.2 | 22 | 14 |  |  |  |  |  |
| GA_53_ | 2 | 5.65 | + | 6 | 347.2>329.2 | 48 | 22 |  |  |  |  |  |

**Table S2. Hormone levels in 23-day-old roots of 13 Arabidopsis accessions (unit: pg mg^-1^ dry weight)**

|  | IAA | | | ABA | | | iP | | | iPR | | | cZ | | | tZ | | | tZR | | | tZ_7_G | | | tZ(O,_9_)G | | | GA_9_ | | |
| --- | --- | --- | --- | --- | --- | --- | --- | --- | --- | --- | --- | --- | --- | --- | --- | --- | --- | --- | --- | --- | --- | --- | --- | --- | --- | --- | --- | --- | --- | --- |
| An-1 | 385.8 | ± | 11.8 | 22.3 | ± | 2.6 | 14.1 | ± | 0.5 | 10.3 | ± | 0.9 | 60.2 | ± | 2.6 | 21.5 | ± | 0.6 | 50.0 | ± | 9.2 | 194.3 | ± | 10.4 | 307.8 | ± | 7.9 | 107.6 | ± | 28.9 |
| Bay-0 | **504.9** | **±** | **14.2** | 17.5 | ± | 2.0 | 15.1 | ± | 0.1 | **21.8** | **±** | **0.8** | 55.7 | ± | 1.4 | 21.2 | ± | 0.4 | 64.4 | ± | 6.5 | 148.4 | ± | 10.4 | 240.5 | ± | 16.7 | 108.9 | ± | 14.1 |
| Bor-4 | 396.4 | ± | 19.4 | 16.2 | ± | 1.1 | 14.1 | ± | 0.3 | 19.4 | ± | 1.2 | 53.9 | ± | 4.4 | 20.9 | ± | 0.4 | 44.5 | ± | 2.9 | 178.6 | ± | 11.1 | 269.4 | ± | 13.2 | **68.4** | **±** | **2.5** |
| Bur-0 | **318.2** | **±** | **35.6** | 16.0 | ± | 2.6 | 14.3 | ± | 0.2 | 13.0 | ± | 0.8 | 58.1 | ± | 1.4 | **20.4** | **±** | **0.3** | 46.6 | ± | 2.5 | 199.2 | ± | 13.7 | 287.8 | ± | 26.8 | **209.0** | **±** | **56.0** |
| Col-0 | 387.6 | ± | 2.2 | 15.5 | ± | 0.6 | 13.8 | ± | 0.4 | 13.7 | ± | 2.1 | 53.2 | ± | 6.9 | 22.3 | ± | 0.2 | 50.8 | ± | 4.3 | 140.7 | ± | 17.9 | 229.2 | ± | 10.5 | 105.2 | ± | 23.2 |
| Cvi-0 | 377.7 | ± | 18.7 | **28.0** | **±** | **2.0** | **13.4** | **±** | **0.9** | 10.0 | ± | 1.0 | 58.7 | ± | 3.7 | **32.0** | **±** | **4.0** | **37.1** | **±** | **5.9** | **138.2** | **±** | **10.0** | **212.5** | **±** | **10.5** | 142.9 | ± | 23.1 |
| Est-1 | 468.6 | ± | 6.1 | 16.3 | ± | 1.1 | 13.8 | ± | 0.3 | 13.8 | ± | 1.4 | 52.1 | ± | 4.3 | 21.1 | ± | 0.6 | 41.8 | ± | 4.2 | 164.9 | ± | 6.6 | 300.5 | ± | 18.4 | 139.8 | ± | 11.4 |
| Fei-0 | 410.9 | ± | 14.2 | **15.2** | **±** | **1.1** | 14.6 | ± | 0.1 | 12.0 | ± | 0.5 | **46.7** | **±** | **5.4** | 21.4 | ± | 0.7 | 41.5 | ± | 0.3 | 145.7 | ± | 4.8 | 265.4 | ± | 26.7 | 121.5 | ± | 19.1 |
| Ler-0 | 457.6 | ± | 6.0 | 16.6 | ± | 1.0 | 15.2 | ± | 0.3 | 17.0 | ± | 2.3 | 74.3 | ± | 2.8 | 21.5 | ± | 0.4 | 48.6 | ± | 2.8 | 191.4 | ± | 11.8 | 305.4 | ± | 9.8 | 95.0 | ± | 23.0 |
| RRS-7 | 329.0 | ± | 4.1 | 16.0 | ± | 2.1 | 15.4 | ± | 0.6 | **9.5** | **±** | **1.9** | 63.4 | ± | 5.2 | 24.2 | ± | 1.2 | 47.1 | ± | 4.4 | **212.7** | **±** | **26.0** | 314.0 | ± | 23.7 | 168.6 | ± | 28.8 |
| Sha-0 | 381.1 | ± | 15.7 | 20.4 | ± | 2.3 | 15.1 | ± | 0.3 | 18.1 | ± | 1.0 | 87.8 | ± | 2.0 | 23.2 | ± | 0.6 | **78.8** | **±** | **10.6** | 192.5 | ± | 22.1 | **339.7** | **±** | **13.8** | 178.3 | ± | 5.6 |
| Ts-1 | 377.2 | ± | 28.7 | 17.0 | ± | 2.1 | **15.7** | **±** | **0.5** | 11.6 | ± | 0.8 | 61.1 | ± | 5.9 | 22.0 | ± | 0.8 | 42.1 | ± | 3.4 | 142.0 | ± | 16.1 | 244.8 | ± | 15.3 | 172.3 | ± | 30.0 |
| Tsu-0 | 360.5 | ± | 8.9 | 20.1 | ± | 4.0 | 14.9 | ± | 0.2 | 16.5 | ± | 1.6 | **90.9** | **±** | **1.9** | 22.0 | ± | 0.2 | 61.1 | ± | 2.0 | 208.5 | ± | 9.8 | 307.9 | ± | 26.4 | 104.9 | ± | 11.7 |
| Median | 391.8 | | | 16.4 | | | 14.7 | | | 13.5 | | | 58.7 | | | 21.9 | | | 46.8 | | | 172.1 | | | 286.8 | | | 120.5 | | |
| Mean | 402.7 | | | 17.8 | | | 14.6 | | | 14.7 | | | 63.1 | | | 22.2 | | | 50.7 | | | 174.1 | | | 280.7 | | | 126.7 | | |
| S.D | 57.6 | | | 4.3 | | | 0.9 | | | 4.4 | | | 14.8 | | | 2.6 | | | 13.7 | | | 34.1 | | | 45.1 | | | 49.8 | | |

Bolded figures indicate the lowest and highest values of each hormone.

**Table S3. Contribution of variables (hormones and root phenotypic traits) on PCA (unit: %)**

| variables | PC1 | PC2 | PC3 | PC4 | PC5 |
| --- | --- | --- | --- | --- | --- |
| RFW | 5.07 | 0.68 | 4.07 | 0.04 | 2.45 |
| MRUN | 4.20 | 4.48 | 2.45 | 0.32 | 1.28 |
| TRL | 7.26 | 0.50 | 0.95 | 0.48 | 1.36 |
| TRTN | 6.16 | 1.54 | 3.03 | 0.63 | 0.49 |
| TRD | 2.34 | 0.12 | 12.94 | 0.20 | 4.89 |
| RL | 3.50 | 5.66 | 3.33 | 0.83 | 2.72 |
| LRL | 7.77 | 0.08 | 0.07 | 0.90 | 0.41 |
| LRN | 4.79 | 0.15 | 8.40 | 1.11 | 0.35 |
| LRD | 1.79 | 8.45 | 4.41 | 0.26 | 4.55 |
| 2′-LRL | 5.55 | 2.50 | 2.88 | 0.20 | 1.89 |
| 2′-LRN | 5.43 | 3.18 | 1.13 | 2.05 | 0.42 |
| 2′-LRD | 0.46 | 2.71 | 10.31 | 5.54 | 1.98 |
| LRL-1Q | 6.07 | 1.46 | 0.18 | 0.05 | 6.85 |
| LRL-2Q | 7.06 | 0.11 | 0.56 | 2.29 | 0.01 |
| LRL-3Q | 3.90 | 6.85 | 0.38 | 3.24 | 1.34 |
| LRL-4Q | 0.29 | 13.02 | 0.73 | 0.03 | 2.42 |
| LRN-1Q | 2.27 | 1.48 | 3.26 | 11.65 | 0.01 |
| LRN-2Q | 4.38 | 0.73 | 2.61 | 5.92 | 0.31 |
| LRN-3Q | 4.32 | 0.37 | 10.08 | 0.10 | 0.01 |
| LRN-4Q | 0.79 | 8.05 | 5.85 | 5.93 | 0.30 |
| IAA | 0.36 | 3.10 | 0.27 | 4.09 | 25.55 |
| ABA | 0.01 | 2.21 | 0.87 | 14.09 | 10.86 |
| iP | 0.19 | 3.37 | 2.54 | 8.51 | 5.09 |
| iPR | 3.32 | 0.28 | 5.61 | 0.70 | 10.00 |
| cZ | 2.34 | 10.06 | 1.13 | 0.39 | 0.97 |
| tZ | 1.25 | 2.28 | 3.59 | 13.72 | 3.06 |
| tZR | 3.60 | 5.27 | 1.82 | 0.01 | 3.08 |
| tZ_7_G | 2.99 | 1.76 | 0.08 | 4.07 | 7.20 |
| tZ(O_, 9_)G | 1.99 | 3.68 | 0.03 | 3.71 | 0.04 |
| GA_9_ | 0.41 | 5.76 | 6.31 | 8.80 | 0.01 |

**Figure S1. Illustration of hydroponic culture**

**
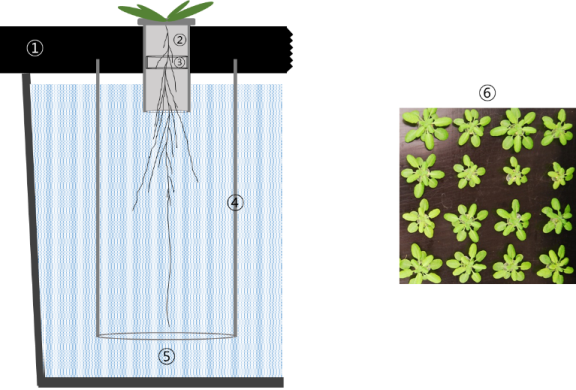
**

The black plate^①^ is placed and covered on the hydroponic tank. A sprout on the top of holder-tube (0.5 ml)^②^ filled with 0.5 % agar in half of Hoagland’s nutrient solution, grows and develops root system in the hydroponic tank. A small ring^③^ inside the tube was tightly placed to prevent agar medium from being slipped down out of the tube during culture, with no physical hindrance for root development. In order to avoid roots to be entangled with roots of neighbour plants, a polypropylene column (diameter 3cm height 5.5 cm)^④^ was equipped underneath the black plate, allowing nutrient solution and roots not to be blocked on the bottom^⑤^. Right picture^⑥^ shows how upper shoots settle and grow on the black plate.

**Figure S2. Hierarchical cluster analysis of RSA traits in 13 Arabidopsis accessions**

| 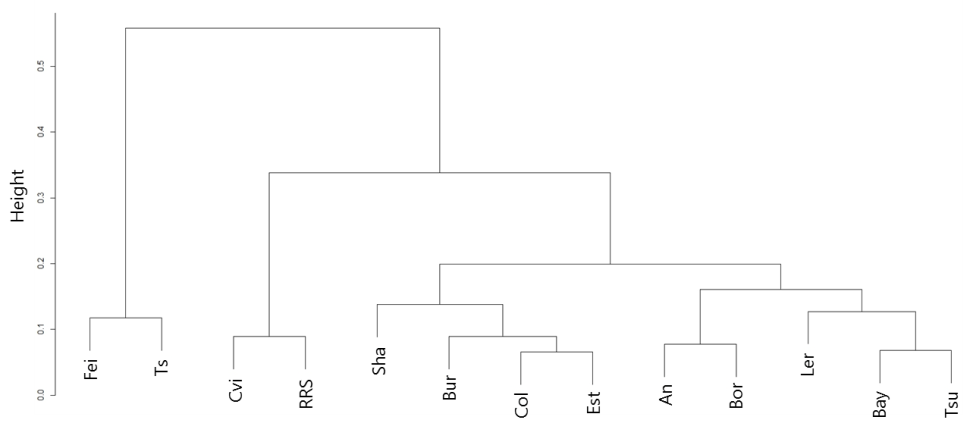 |
| --- |

**Figure S3. Dry root weights of Col-0 and Ler-0 during five weeks of root development**

| 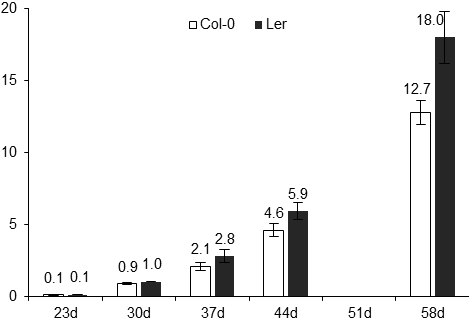 Days  mg / root |
| --- |

Data of 51th day was missed.

**Figure S4. Re-partition of 13 Arabidopsis accession in PCA of hormone levels and RSA traits**

|  |
| --- |
